# *Salmonella* Enteritidis activates inflammatory storm *via* SPI-1 and SPI-2 to promote intracellular proliferation and bacterial virulence

**DOI:** 10.3389/fcimb.2023.1158888

**Published:** 2023-05-30

**Authors:** Dan Xiong, Li Song, Yushan Chen, Xinan Jiao, Zhiming Pan

**Affiliations:** ^1^ Jiangsu Key Laboratory of Zoonosis, Yangzhou University, Yangzhou, China; ^2^ Jiangsu Co-Innovation Center for Prevention and Control of Important Animal Infectious Diseases and Zoonoses, Yangzhou University, Yangzhou, China; ^3^ Key Laboratory of Prevention and Control of Biological Hazard Factors (Animal Origin) for Agrifood Safety and Quality, Ministry of Agriculture and Rural Affairs, Yangzhou University, Yangzhou, China

**Keywords:** *Salmonella* Pathogenicity Island, Inflammatory pathway, bacterial colonization, macrophage, mouse infection model

## Abstract

*Salmonella* Enteritidis is an important intracellular pathogen, which can cause gastroenteritis in humans and animals and threaten life and health. *S.* Enteritidis proliferates in host macrophages to establish systemic infection. In this study, we evaluated the effects of *Salmonella* pathogenicity island-1 (SPI-1) and SPI-2 to *S.* Enteritidis virulence *in vitro* and *in vivo*, as well as the host inflammatory pathways affected by SPI-1 and SPI-2. Our results show that *S.* Enteritidis SPI-1 and SPI-2 contributed to bacterial invasion and proliferation in RAW264.7 macrophages, and induced cytotoxicity and cellular apoptosis of these cells. *S.* Enteritidis infection induced multiple inflammatory responses, including mitogen-activated protein kinase (ERK-mediated) and Janus kinase-signal transducer and activator of transcript (STAT) (STAT2-mediated) pathways. Both SPI-1 and SPI-2 were necessary to induce robust inflammatory responses and ERK/STAT2 phosphorylation in macrophages. In a mouse infection model, both SPIs, especially SPI-2, resulted in significant production of inflammatory cytokines and various interferon-stimulated genes in the liver and spleen. Activation of the ERK- and STAT2-mediated cytokine storm was largely affected by SPI-2. *S.* Enteritidis *ΔSPI-1*-infected mice displayed moderate histopathological damage and drastically reduced bacterial loads in tissues, whereas only slight damage and no bacteria were observed in *ΔSPI-2-* and *ΔSPI-1/SPI-2*-infected mice. A survival assay showed that *ΔSPI-1* mutant mice maintained a medium level of virulence, while SPI-2 plays a decisive role in bacterial virulence. Collectively, our findings indicate that both SPIs, especially SPI-2, profoundly contributed to *S.* Enteritidis intracellular localization and virulence by activating multiple inflammatory pathways.

## 1 Introduction

*Salmonella enterica* is a Gram-negative and facultative intracellular pathogen that can lead to various diseases, such as gastroenteritis and even lethal systemic infections, in human and animal hosts (Kurtz et al., 2017; Grenz et al., 2018). Salmonellosis is usually caused by contaminated food and water, and is the most common foodborne disease in humans, especially in developing countries with substandard water supplies and poor sanitation (Parry et al., 2002; Wain et al., 2015). In March 2022, a multi-country outbreak of *Salmonella* was linked to chocolate products. In total, 151 genetically related cases of *Salmonella* suspected to be associated with the consumption of the implicated chocolate products were reported in 11 countries (WHO, 2022). The *Salmonella* genus contains six subspecies and more than 2600 serovars, most of which are pathogenic to humans and animals (LeLièvre et al., 2019). Two major salmonellosis-causing pathogens, *S.* Typhimurium and *S.* Enteritidis, are responsible for more than 1 million infections in the U.S. every year (CDC, 2023).

*Salmonella* pathogenicity islands (SPIs) are large regions of bacterial DNA encoding *Salmonella* virulence factors. The type III secretion systems (T3SSs) encoded by SPI-1 and SPI-2 are complicated machineries that play crucial roles for *Salmonella* pathogenesis (Que et al., 2013). *Salmonella* SPIs allow the transportation of effector proteins into the host cytoplasm, playing particularly important functions during *Salmonella* infection (Srikanth et al., 2011; Jennings et al., 2017). The SPI-1 gene cluster is a 40-kb region, and its encoded 39 genes include T3SS-1 and effector proteins, as well as the transcription regulatory factors that modulate the expression of numerous virulence genes inside and outside SPI-1 (Hansen-Wester and Hensel, 2001; Zhang et al., 2018). Comparative genomic analysis showed that the SPI-2 T3SS effector repertoire was very different among different *Salmonella* serovars, with subtle variations infrequently occurring between strains of the same serovar (Nuccio and Bäumler, 2014).

Previous studies of individual *Salmonella* effectors revealed a series of complex and diverse new biochemical activities. These effectors frequently display exquisite specificity and potency in subverting, avoiding, or reprogramming host cellular processes (Keszei et al., 2014; Rolhion et al., 2016; Paul et al., 2022). Different effector proteins of SPI-1 and SPI-2 could stimulate or inhibit innate immune responses (Jennings et al., 2017). Macrophages usually represent the first line of host defenses against bacterial pathogens and are vital niches in the pathogenic process of *Salmonella* (Jiang et al., 2021). Cytokines play an important role in host-pathogen intervention by inducing the release of either pro- or anti-inflammatory signaling molecules to regulate immune responses (Paul et al., 2022). However, a prolonged or excessive cytokine storm underlies many chronic inflammatory diseases and can be lethal to the host (O'Neill et al., 2009; Kawai and Akira, 2010; Brandes et al., 2013).

Previous studies investigating the transmission and colonization of *Salmonella* have focused on *S.* Typhimurium (Lee et al., 2005; Rana and Kulshreshtha, 2006; Jones et al., 2007). Molecular analysis revealed how *S.* Typhimurium manipulates host cell signaling through effector proteins. Recent studies show that *S.* Enteritidis is the most prevalent serovar among *Salmonella* isolates from children with diarrhea (Zhao et al., 2022). However, the contribution and related signaling pathways of SPI-1 and SPI-2 in inflammation caused by *S.* Enteritidis during the course of infection remain unclear. Previous studies investigated the functions of SPI-1 and SPI-2 mainly through use of genetic mutant strains; for example, *ssaRmut* and *invAmut* representing mutations of SPI-1 and SPI-2, respectively (Sekirov et al., 2010). However, the effects of *S.* Enteritidis in the context of complete SPI-1 and SPI-2 deficiency have rarely been studied. Moreover, although SPI participates in *Salmonella* virulence, whether it is directly involved in activation of other signaling pathways, such as Janus kinase-signal transducer and activator of transcript (JAK-STAT), remains to be understood.

In this study, to evaluate the contribution of *S.* Enteritidis SPIs on the inflammatory pathways, bacterial colonization and virulence in systemic infection, we evaluated the effects of *S.* Enteritidis SPI-1 and SPI-2 to bacterial invasion of the host cellular cytosol, intracellular survival, and induction of cellular apoptosis. The host inflammatory pathways and bacterial virulence affected by SPI-1 and SPI-2 were also determined in macrophages and in a mouse infection model. This study will improve understanding of the molecular mechanisms employed by different *Salmonella* SPIs to promote the bacterial survival during infection of the host.

## 2 Materials and methods

### 2.1 Mice and ethics statement

Specific pathogen-free C57BL/6J mice (female, 6–8 weeks) were purchased from Beijing Vital River Laboratory Animal Technology (Beijing, China). Mice were raised in an environment with a light/dark cycle of 12 h, a constant temperature of 25°C and a humidity of 50%. A standard chow diet was provided for mice *ad libitum* throughout the experiments. All animal studies were conducted according to protocols approved by the Committee on the Ethics of Animal Experiments of Yangzhou University [Approval ID: SYXK (Su) 2017-0044].

### 2.2 Bacterial strains and cell line

Bacterial strains and plasmids used in the present study are shown in **Supplementary Table 1**. The *S.* Enteritidis strain CMCC50041 (GenBank Accession No. CP013097.1) was used as the wild-type (WT) strain and for the construction of SPI-1 and SPI-2 mutants. SPI-1 and SPI-2 mutants of *S.* Enteritidis were previously constructed (Xiong et al., 2019). The *ΔSPI-1/SPI-2* double-deficient strain in this study was constructed following the λ-Red recombinase gene replacement method (Datsenko and Wanner, 2000; Jiang et al., 2017). Primer sequences to generate and confirm the mutant strains are listed in **Supplementary Table 2**. All bacterial strains were cultured on Luria-Bertani (LB) agar plates or in LB broth with necessary antibiotics at appropriate concentrations (e.g., 100 μg/mL ampicillin and 20 μg/mL chloramphenicol).

The murine RAW264.7 macrophage cell line was provided by the Shanghai Institute of Biochemistry and Cell Biology of the Chinese Academy of Sciences (Shanghai, China). Cells were maintained in Dulbecco’s Modified Eagle Medium (DMEM) supplemented with 10% fetal bovine serum (FBS) and 1% penicillin-streptomycin (Gibco, Waltham, MA, USA) at 37°C in a 5% CO_2_ incubator.

### 2.3 Construction of mutant strains using the λ-red system

The λ-Red system, an effective and widely used approach to inactivate the chromosomal genes in *Escherichia coli* and *S.* Typhimurium (Tischer et al., 2006), was used to produce SPI-1 and SPI-2 deficient mutants. *ΔSPI-1/SPI-2* double mutant strain was constructed using *ΔSPI-2* forward and reverse primers based on the *ΔSPI-1* deficient strain. All gene deletions were verified by PCR analysis and sequencing. All primers used for amplification of certain regions in SPI-1 and SPI-2 of *S.* Enteritidis are shown in **Supplementary Table 2**. Growth curves of *S.* Enteritidis C50041 WT, *ΔSPI-1*, *ΔSPI-2*, and *ΔSPI-1/SPI-2* deletion mutants were determined to evaluate the influence of SPI deficiency on *Salmonella* growth. Biochemical tests were performed using an API 20E identification kit (bioMerieux SA, Lyon, France) in accordance with the manufacturer’s protocol.

### 2.4 *S.* Enteritidis infection of macrophages

*S.* Enteritidis colonies were cultured overnight at 37°C with agitation (180 rpm) in LB liquid broth. Log-phase bacteria were obtained by diluting the stationary-phase bacteria to fresh LB at a ratio of 1: 40 and subculturing to the mid-log-phase (3 h). Bacteria were pelleted, washed three times with cold phosphate-buffered saline (PBS), and resuspended with DMEM. The bacterial concentration was estimated based on the optical density at 600 nm, and the CFUs were calculated by serial dilution on agar plates. Before infection, RAW264.7 macrophages were seeded in 24‐well plates at a density of 2 × 10^5^ cells/well overnight in DMEM containing 10% FBS without antibiotics to obtain 80% confluence. Next, *Salmonella* was added at a multiplicity of infection of 100:1. Plates were centrifuged at 1,000 × g for 8 min to synchronize infection and allowed to invade for 1 h. After washing cells twice with DPBS, DMEM supplemented with 100 μg/mL gentamicin was used to kill the extracellular bacteria for 1 h.

### 2.5 Invasion and proliferation assays

For invasion assays, RAW264.7 cells continued to be infected for 0.5 h, washed twice with DPBS, and lysed with 1% Triton X-100. Subsequently CFU numbers of intracellular bacteria were calculated to determine the bacterial invasion by plating on LB agar. For the proliferation assay, cells continued to be infected for 0.5 h, washed twice with DPBS, and continued to be maintained in culture medium containing 10 μg/mL gentamicin for 2, 4, 6, or 8 h to quantify bacterial proliferation.

### 2.6 Cytotoxicity and apoptosis analysis

Murine RAW264.7 macrophages were seeded in 24-well plates at a density of 2 × 10^5^ cells per well and infected with *Salmonella* WT and mutants as described above. Cells cultured in DMEM were set as the negative control group, which was not infected with bacteria. After *Salmonella* infection for 2 h, culture supernatants were harvested for the quantification of lactate dehydrogenase (LDH) with an LDH Cytotoxicity Assay Kit (Beyotime, Shanghai, China). The absorbance value of the DMEM control group was subtracted from all treatment groups according to the manufacturer’s instructions. In addition, cells were collected and centrifuged at 1,000 × g for 10 min and resuspended in 400 μL of binding buffer. Early and late apoptosis among different groups were conducted using an Annexin V-FITC/PI Apoptosis Detection Kit (Vazyme, Nanjing, China). Percentages of apoptotic cells were analyzed using a FACS Aria flow cytometer (BD Biosciences, Franklin Lakes, NJ, USA) with FlowJo_v10 software (FowJo, Ashland, OR, USA).

### 2.7 *In vivo* mouse infection models

Female C57BL/6J mice were randomly divided into 5 groups (*n* = 13 per group, *n* = 5 for tissue collection and *n* = 8 for survival monitoring). For oral infections, water and food were withdrawn for 4 h before C57BL/6J mice were infected intragastrically with 10^6^ CFU of *S.* Enteritidis C50041 WT or the indicated *ΔSPI-1*, *ΔSPI-2*, or *ΔSPI-1/SPI-2*-deficient strains in a 200-μL volume. Bacteria were prepared and CFU numbers were determined as described above. Drinking water and food were offered at 2 h post infection. To evaluate the cytokine levels in livers and spleens of mice, tissues were homogenized using a Precellys 24 homogenizer (Rockville, MD, USA). Cytokine expression levels in organs were measured using qRT-PCR. To evaluate the activation of MAPK and JAK-STAT signaling pathways in the tissues, homogenized specimens were centrifuged for 10 min at 12,000 × g at 4°C. p-ERK and p-STAT2 levels in collected supernatants were measured by Western blotting. For analysis of the bacterial burden in tissues, infected mice were sacrificed 4 days post infection. Next, mouse cecums, livers and spleens were aseptically collected, homogenized in sterile ice-cold PBS, serially ten‐fold diluted, and plated on LB plates for CFU determination. Before homogenization, the cecums were aseptically removed and rinsed three times with sterile PBS. Meanwhile, tissue samples from the spleen and liver were removed for pathological analysis. The mortality of mice in different infection groups was recorded daily and monitored for 2 weeks.

### 2.8 Western blotting analysis

Cells were collected and lysed in Cell Lysis Buffer for Western (Beyotime) followed by centrifugation at 12,000 × g at 4°C for 5 min. The supernatant was harvested and the protein concentration was determined by BCA Protein Assay Kit (Beyotime). Protein samples were subjected to 12% sodium dodecyl sulfate-polyacrylamide gel electrophoresis and the polyvinylidene fluoride membrane was used for protein transfer. The membrane was blocked with 1% bovine serum albumin (BSA) in Tris-buffered saline containing 0.05% Tween 20 (TBST) for 2 h at room temperature, followed by primary antibodies and horseradish peroxidase (HRP)-conjugated IgG secondary antibodies (1:5000 dilution). Antibodies used in this study were listed below: rabbit anti-p-STAT2 (Y690) (Abcam, Cambridge, UK), rabbit monoclonal anti-p-ERK1&2 (Thr202/Tyr204) (Cell Signaling Technology, Danvers, MA, USA), mouse monoclonal anti-β-actin (C4) (Santa Cruz Biotechnology, Dallas, TX, USA), HRP-conjugated goat anti-rabbit IgG H&L (Abcam), and goat anti-mouse IgG H&L-HRP (Calbiochem, San Diego, CA, USA). The immunoblotting was conducted with the SuperSignal™ West Pico PLUS Chemiluminescent Substrate (Thermo Fisher Scientific, Waltham, MA, USA). Images were captured with the Amersham™ Imager 600 System (GE Healthcare, Chicago, IL, USA). Band densities were analyzed using ImageJ software (https://imagej.nih.gov/ij/) and normalized to the corresponding internal marker β-actin.

### 2.9 RNA isolation and quantitative real-time PCR

Total mRNA preparations of RAW264.7 macrophage and mouse tissues were extracted with a Total RNeasy Plus Mini Kit (Qiagen, Hilden, Germany). For preparation of tissue mRNA, mouse livers and spleens were harvested and stored using liquid nitrogen. The frozen tissues were homogenized in RLT buffer before extraction. RNA concentrations were measured using a NanoDrop 1000 spectrophotometer (NanoDrop Technologies, Wilmington, DE, USA). cDNA was synthesized using a PrimeScript RT Reagent Kit (Takara, Dalian, China). qRT-PCR was performed with a QuantStudio 6 Real-Time PCR System (Applied Biosystems, Foster, CA, USA). The total volume of PCR reaction was 20 μL, and it was conducted in triplicate using the Fast Start Universal SYBR Green Master (Roche, Basel, Switzerland) according to the manufacturer’s protocols. The real-time PCR efficiencies (*E*) for the reference gene and target genes were determined using the diluted cDNA templates, and calculated based on the equation: *E* = 10^[–1/slope]^ (Rasmussen, 2001). Normalized levels of gene expression were calculated following the Pfaffl method based on *E* and the CT deviation of the treatment sample versus the control, and expressed in comparison to the reference gene (Pfaffl, 2001). β-actin gene was used as the reference control. Primer sequences for the qRT-PCR are shown in **Supplementary Table 3**.

### 2.10 Immunofluorescence staining

Mouse livers and spleens were collected, formalin-fixed, and embedded in paraffin. The sections were deparaffinized, antigen-retrieved with sodium citrate buffer, permeabilized in Triton X-100 (0.2%) for 0.5 h, and then blocked with PBS containing 5% BSA for 1 h. Mouse p-ERK1&2 and p-STAT2 were stained using rabbit monoclonal anti-p-ERK1&2 and rabbit anti-p-STAT2 (Y690) antibodies with the blocking buffer overnight at 4°C. Cy3-conjugated goat anti-rabbit secondary antibody (Abcam) was diluted in blocking buffer and incubated for 1 h at room temperature according to the manufacturer’s instructions. The nuclear DNA was stained with 6-Diamidino-2-phenylindole (DAPI). The sections were mounted with the coverslips and Prolong Diamond Antifade Mountant (Thermo Fisher Scientific). Images were captured using the fluorescence microscope (Leica, Wetzlar, Germany).

### 2.11 Histopathological evaluation

Mouse tissues were fixed with 10% neutral formalin for 24 h, then embedded with paraffin, cut into sections (5 μm), and stained using hematoxylin and eosin (H&E) by standard techniques. Histology sections were observed using an Eclipse Ci-L microscope (Nikon, Tokyo, Japan). Pathological assessment of livers and spleens were performed by two pathologists in a blinded manner using the criteria described in **Supplementary Table 4**. The sum of the individual scores for each tissue sample was determined as the combined pathological score.

### 2.12 Statistical analysis

Data were comparatively analyzed with the unpaired Student’s *t* test and were shown as mean ± SEM. All statistical analysis was performed with GraphPad Prism (version 8.0.2, GraphPad Software, San Diego, CA, USA). The significant differences were determined when the P-value was ≤ 0.05 (*), 0.01 (**), and 0.001 (***).

## 3 Results

### 3.1 Characteristics of *S.* Enteritidis *ΔSPI-1*, *ΔSPI-2*, and *ΔSPI-1/SPI-2*-deficient strains

**Figure 1:**
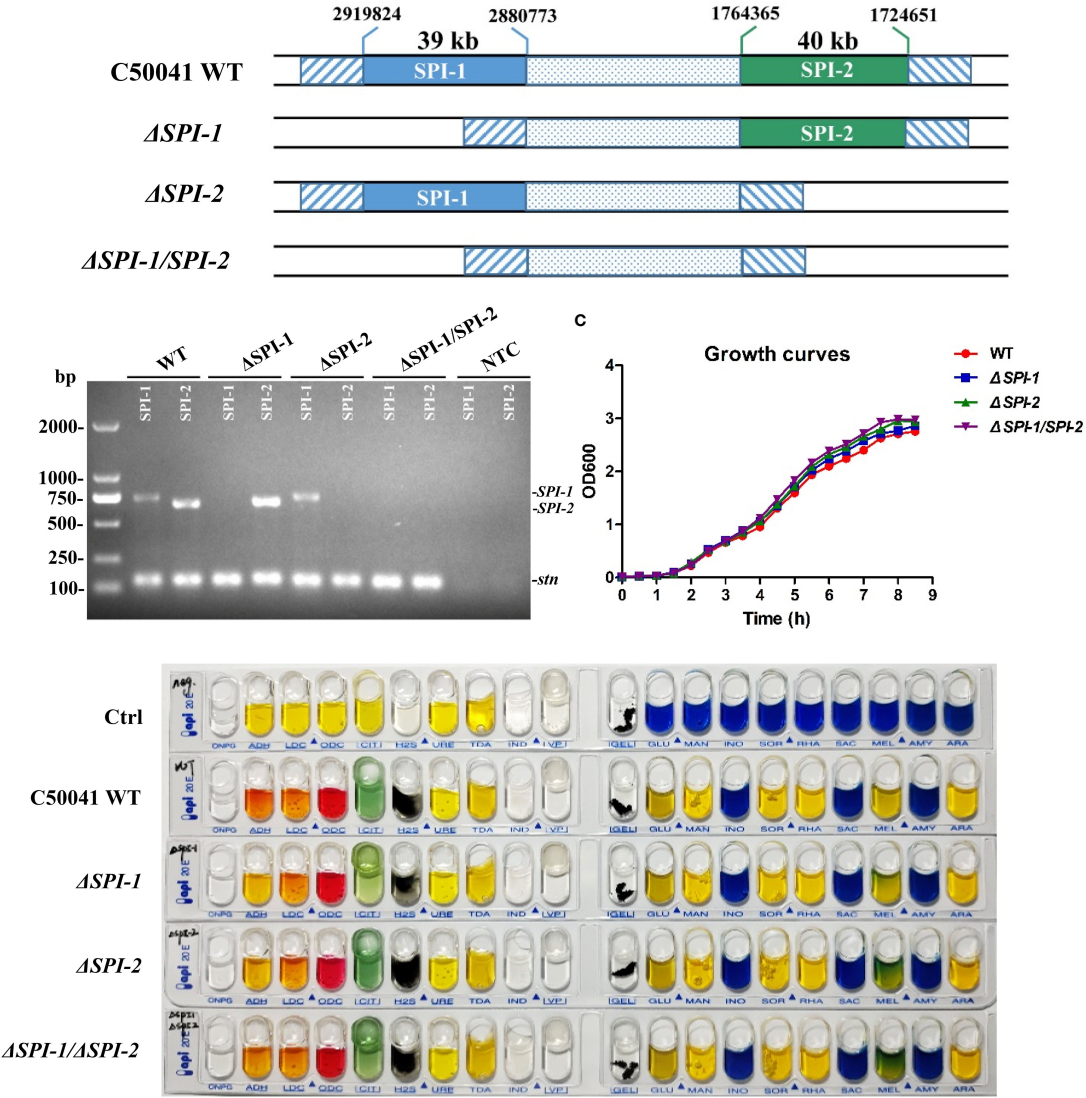
Construction of *S.* Enteritidis *ΔSPI-1*, *ΔSPI-2* and *ΔSPI-1/SPI-2* deficient mutants and biochemical identification of the deficient strains. **(A)** Schematic representation of the construction for *S.* Enteritidis *ΔSPI-1*, *ΔSPI-2* and *ΔSPI-1/SPI-2* mutants. The *Salmonella* deficient mutants were produced by using the λ-Red recombinase gene replacement method. **(B)** All gene deletions were verified by PCR analysis and by sequencing. The *stn* gene was used as the reference control. **(C)** Growth curves of *S.* Enteritidis C50041 wild-type, *ΔSPI-1*, *ΔSPI-2* and *ΔSPI-1/SPI-2* deletion strains. Bacteria were cultured in LB medium at 37°C with 180 rpm, and the OD_600_ values of bacterial cultures were determined in 0.5 h intervals. **(D)** Biochemical tests of *S.* Enteritidis WT, *ΔSPI-1*, *ΔSPI-2* and *ΔSPI-1/SPI-2* strains using the API 20E identification kit. Sterile water was used as control.

*Salmonella ΔSPI-1*, *ΔSPI-2*, and *ΔSPI-1/SPI-2*-deficient strains were constructed to identify roles for each SPI in bacterial virulence of macrophages and a mouse model. Using the λ-Red system, SPI-1 (39 kb) and SPI-2 (40 kb) were deleted to generate *ΔSPI-1* and *ΔSPI-2*, respectively, while both gene cassettes were deleted to generate the *ΔSPI-1/SPI-2*-deficient strain (**Figure 1A**). Each strain was confirmed by PCR analysis and sequencing, which showed that mutants were constructed correctly (**Figure 1B**). Our assessment of growth rates confirmed that bacterial growth was unaffected by deletion of SPI-1 or SPI-2 (**Figure 1C**). In addition, mutant strains were biochemically characterized using an API 20E identification kit, which revealed no differences between mutant strains relative to the WT strain (**Figure 1D**).

### 3.2 Deletion of either *SPI-1* or *SPI-2* reduced *S.* Enteritidis intracellular localization, cytotoxicity, and apoptosis in macrophages

**Figure 2:**
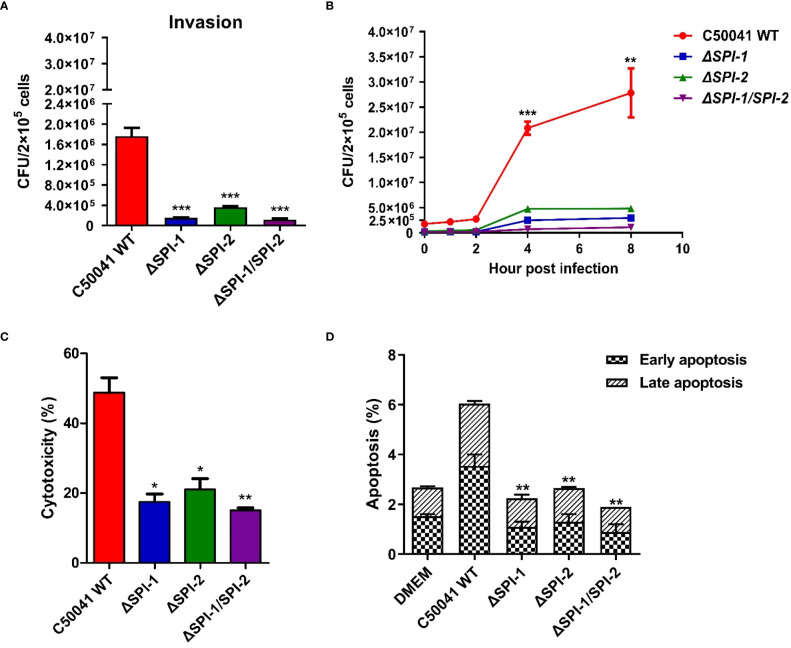
Intracellular localization and virulence evaluation of *S.* Enteritidis *ΔSPI-1*, *ΔSPI-2* and *ΔSPI-1/SPI-2* deficient strains in macrophages. **(A)** The invasion was determined in RAW264.7 following the infection of *S.* Enteritidis WT, *ΔSPI-1*, *ΔSPI-2* and *ΔSPI-1/SPI-2* deficient strains. **(B)** The proliferation was determined at different time points in RAW264.7 macrophages following the infection of *S.* Enteritidis WT, *ΔSPI-1*, *ΔSPI-2* and *ΔSPI-1/SPI-2* deficient strains. **(C)** After *Salmonella* infection for 2 h, the supernatants were harvested for the quantification of LDH levels. LDH levels were determined with the LDH Cytotoxicity Assay Kit. **(D)** Apoptosis analysis with the Annexin V-FITC/PI staining and flow cytometry. The cell apoptosis among different groups was determined with the Annexin V-FITC/PI Apoptosis Detection Kit. P < 0.05 (*), P < 0.01 (**) and P < 0.001 (***) were considered statistically significant.

To study the effects of SPI-1 and SPI-2 on bacterial invasion and proliferation *in vitro*, the RAW264.7 macrophage cells were infected with C50041 WT, *ΔSPI-1*, *ΔSPI-2*, or *ΔSPI-1/SPI-2* strains. Mutants showed significantly decreased abilities to invade macrophages relative to WT (**Figure 2A**). In addition, intracellular proliferation of WT *S.* Enteritidis was substantially increased 2 h following infection compared with the three mutants (**Figure 2B**). Compared with WT *S.* Enteritidis, LDH release caused by mutant strains in macrophages was significantly decreased (**Figure 2C**). In addition, both SPIs independently resulted in the apoptosis of murine macrophages following infection (**Figure 2D**).

### 3.3 Both SPI-1 and SPI-2 were necessary to induce inflammation in macrophages

**Figure 3:**
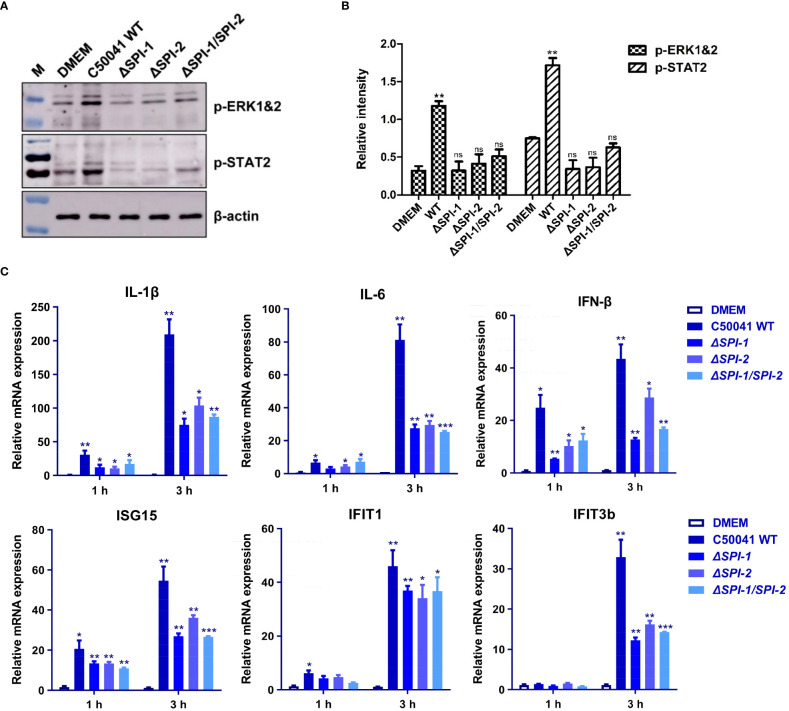
The SPI-1 and SPI-2 promoted *S.* Enteritidis to trigger inflammation storm *via* two different inflammatory pathways in macrophages. **(A)** *Salmonella* SPI-1 and SPI-2 induced the robust activation of MAPK and STAT signaling pathways. Macrophages were infected with *S.* Enteritidis WT, *ΔSPI-1*, *ΔSPI-2* and *ΔSPI-1/SPI-2* deficient strains, and the phosphorylation of ERK and STAT2 was determined. **(B)** Relative intensity of p-ERK and p-STAT2 were semi-quantified using ImageJ and presented as bar graphs. **(C)** *Salmonella* SPI-1 and SPI-2 contributed to significant increase of inflammatory cytokines and ISGs. The RAW264.7 macrophage was infected with *S.* Enteritidis WT, *ΔSPI-1*, *ΔSPI-2* and *ΔSPI-1/SPI-2* deficient strains, and total mRNA was extracted. The expression levels of inflammatory cytokines and various ISGs were determined by using qRT-PCR. Cells cultured in DMEM alone were served as the control group. P < 0.05 (*), P < 0.01 (**) and P < 0.001 (***) were considered statistically significant. ns, not significant.

The effects of SPI-1 and SPI-2 on activation of inflammatory pathways were evaluated in murine macrophages following *Salmonella* infection. The real-time PCR efficiencies for the reference gene and target genes were 2.04 for β-actin, 2.07 for IL-1β, 2.02 for IL-6, 2.15 for IFN-β, 2.08 for ISG15, 2.16 for IFIT1, 1.96 for IFIT2, 1.98 for IFIT3b and 2.05 for CXCL2 (**Supplementary Figure 1**). The results show that SPI-1 and SPI-2 of *S.* Enteritidis induced multiple inflammatory responses, including MAPK (ERK-mediated) and JAK-STAT (STAT2-mediated) pathways (**Figures 3A, B**). Moreover, *S.* Enteritidis SPI-1 and SPI-2 contributed to robust production of inflammatory cytokines such as interleukin (IL)-1β and IL-6, and interferon-stimulated genes (ISGs) such as IFN-β, ISG15, IFIT1, and IFIT3b (**Figure 3C**). Inflammatory responses 3 h after infection were stronger compared with those at 1 h. Collectively, these results demonstrate that both SPI-1 and SPI-2 of *S.* Enteritidis are necessary to induce inflammation in macrophages.

### 3.4 SPI deficiency decreased inflammatory cytokines and immune signaling *in vivo*

**Figure 4:**
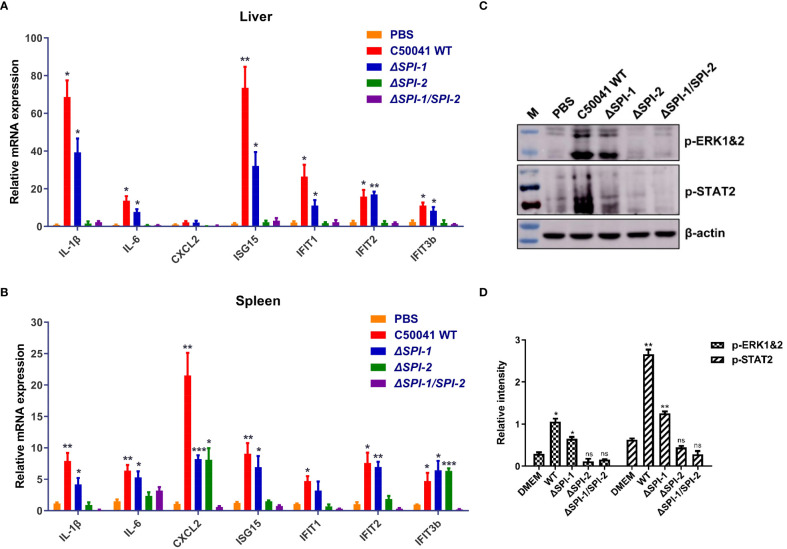
The *Salmonella* SPI-1 and SPI-2 resulted in the cytokine storm in livers and spleens following infection in mice. Inflammatory cytokines and ISGs were determined in livers **(A)** and spleens **(B)** of mice using qRT-PCR. On 4 day following infection, the tissues were harvested and homogenized in RLT buffer for the mRNA extraction. The cytokine expression levels in the organs were measured using qRT-PCR. **(C)** Western blotting analysis of the phosphorylation of ERK- and STAT2-mediated inflammatory pathways in the spleen following *Salmonella* infection. On 4 day following infection, the spleens were homogenized and centrifuged to harvest the supernatant for the immunoblotting analysis. **(D)** Relative intensity of p-ERK and p-STAT2 were semi-quantified using ImageJ and presented as bar graphs. P < 0.05 (*), P < 0.01 (**) and P < 0.001 (***) were considered statistically significant. ns, not significant.

To evaluate the contribution of SPI-1 and SPI-2 to the virulence of *S.* Enteritidis *in vivo*, immune responses following *Salmonella* infection were determined in a mouse model. The results show that deficiency of the two SPIs, especially SPI-2, significantly abolished the production of inflammatory cytokines and various ISGs in liver and spleen (**Figures 4A, B**). In addition, infection with WT *Salmonella* resulted in robust phosphorylation of ERK and STAT2. However, the SPI-1 mutant displayed reduced p-ERK and p-STAT2 levels. Notably, *ΔSPI-2* and *ΔSPI-1/SPI-2*-deficient strains completely abolished the activation of the two inflammatory pathways (**Figures 4C, D**).

### 3.5 *SPI-1* and *SPI-2* contributed to activation of MAPK and STAT signaling in mice

**Figure 5:**
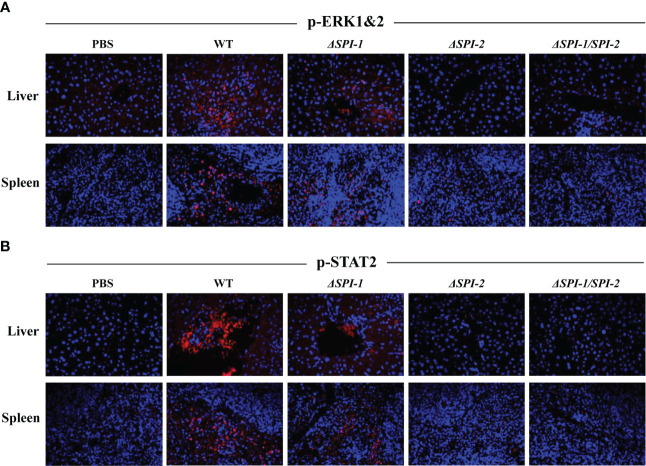
*S.* Enteritidis WT induced the pronounced activation of p-ERK1&2 and p-STAT2 in the livers and spleens of the infected mice. The activation of p-ERK1&2 **(A)** and p-STAT2 **(B)** in mouse liver and spleen were stained using the corresponding antibodies. The nuclear DNA was stained with DAPI. The sections were mounted with the coverslips and Prolong Diamond Antifade Mountant. Images were captured using the Leica confocal microscope at 400 × magnification.

To further characterize the evoked immune response, we performed immunofluorescence assays to examine activation of inflammation-related signaling pathways in the liver and spleen of mice following *Salmonella* infection. Expression of p-ERK1&2 and p-STAT2 was evaluated as markers of the activation of MAPK and STAT signaling pathways. The results show that WT *S.* Enteritidis induced the most pronounced activation of p-ERK1&2 and p-STAT2 in liver and spleen tissues of infected mice, while the SPI-1 mutant induced moderate activation of inflammatory signaling. However, neither *ΔSPI-2*- or *ΔSPI-1/SPI-2*-infected mice displayed obvious inflammation activation, showing similar results to the PBS control group (**Figure 5**).

### 3.6 *SPI-1* and *SPI-2* contributed to cytokine storm and tissue damage

**Figure 6:**
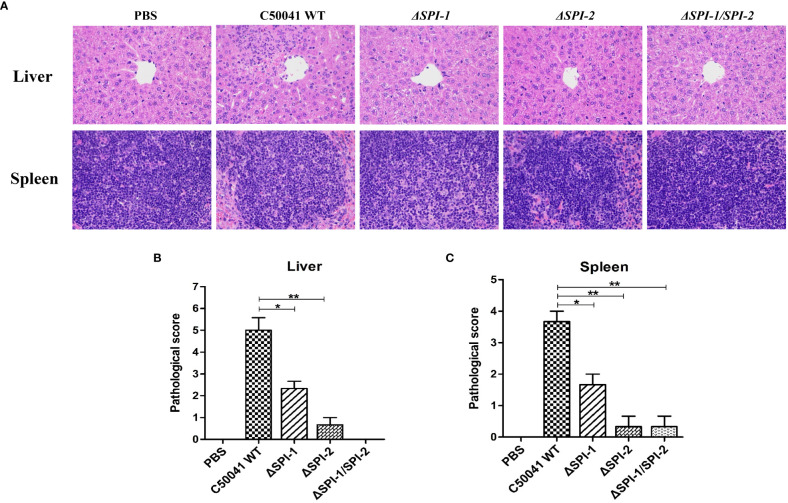
*S.* Enteritidis SPI-1 and SPI-2 contributed to inflammation storm and tissue damages in the mouse infection model. **(A)** Histopathological assessment of the livers and spleens from mice infected with *S.* Enteritidis WT, *ΔSPI-1*, *ΔSPI-2*, and *ΔSPI-1/SPI-2*. Mice were infected with different bacteria, and the livers and spleens were stained with H&E after paraformaldehyde fixation at 4 dpi. Images were captured using a microscope at 400 × magnification. Pathological scores of livers **(B)** and spleens **(C)** were evaluated from mice infected with *Salmonella* C50041, *ΔSPI-1*, *ΔSPI-2*, and *ΔSPI-1/SPI-2*. Pathological evaluations were performed using criteria described in **Supplementary Table 4**. P < 0.05 (*) and P < 0.01 (**) were considered statistically significant.

Pro-inflammatory cytokines activate inflammatory processes that result in macrophage recruitment and polymorphonuclear neutrophil infiltration, which lead to cellular injury in the host. Characteristic signs of this process include strong multifocal neutrophilic infiltration, multifocal necrosis, severe diffusion, and cell swelling. To investigate the impact of SPI on pathological manifestations of liver and spleen injury, we performed histopathologic analyses of *S.* Enteritidis‐infected mice. As shown in **Figure 6**, liver and spleen tissues from mice infected with WT *S.* Enteritidis C50041 exhibited severe inflammation and damage on day 4 post infection. The average pathological scores for liver and spleen of WT-infected mice were 5 and 3.7 with severe neutrophil infiltration and necrosis. *S.* Enteritidis *ΔSPI-1*-infected mice demonstrated moderate histopathological changes in liver and spleen tissues with the average pathological scores of 2.3 and 1.7 respectively as a result of infection. However, tissues from mice infected with *ΔSPI-2* and *ΔSPI-1/SPI-2* mutants displayed slight damage and inflammation similar to that observed in control mice. The average pathological scores for liver and spleen of *ΔSPI-2*-infected mice were 0.7 and 0.3 with mild neutrophil infiltration. Slight inflammation and even no lesions were observed in *ΔSPI-1/SPI-2*-infected mice with the average pathological score of 0.3 for the spleen.

### 3.7 *SPI-1* and *SPI-2* contributed to *S.* Enteritidis virulence in a mouse infection model

**Figure 7:**
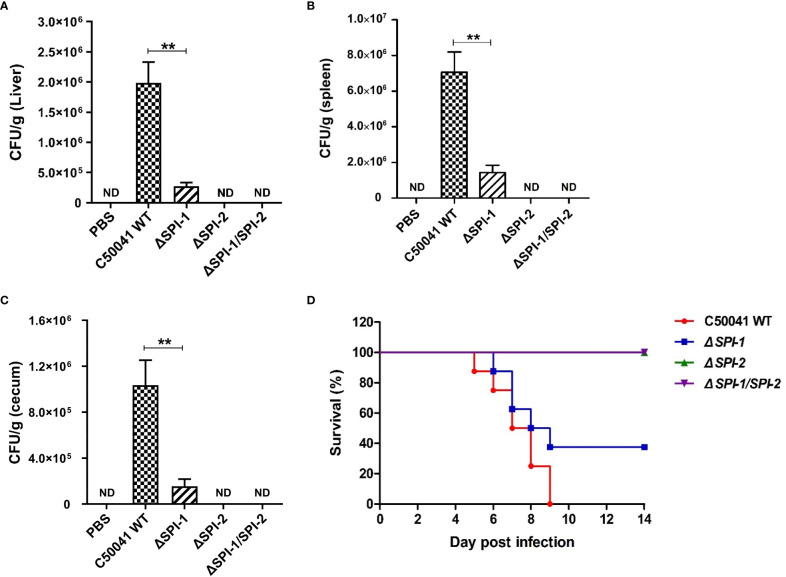
SPI-1 and SPI-2 promoted S. Enteritidis localization in tissues and the bacterial virulence in the mouse infection model. SPI-1 and SPI-2 promoted Salmonella localization in mouse liver **(A)**, spleen **(B)** and cecum **(C)**. The bacterial burden in tissues were determined on day 4 post infection. Mice were sacrificed, and the livers, spleens and cecums were aseptically collected, homogenized with sterile ice-cold PBS, serially 10‐fold diluted, and plated on LB plates for the determination of CFU. **(D)** Percentage survival of mice after infection with C50041, *ΔSPI-1, ΔSPI-2* and *ΔSPI-1/SPI-2*. Mice were fasted for 4 h and given 106 CFU of bacteria in 0.1 mL PBS intragastrically. The mortality of mice in different infection groups was recorded daily and monitored for two weeks. P < 0.01 (**) was considered statistically significant. ND, not detected.

**Figure 8:**
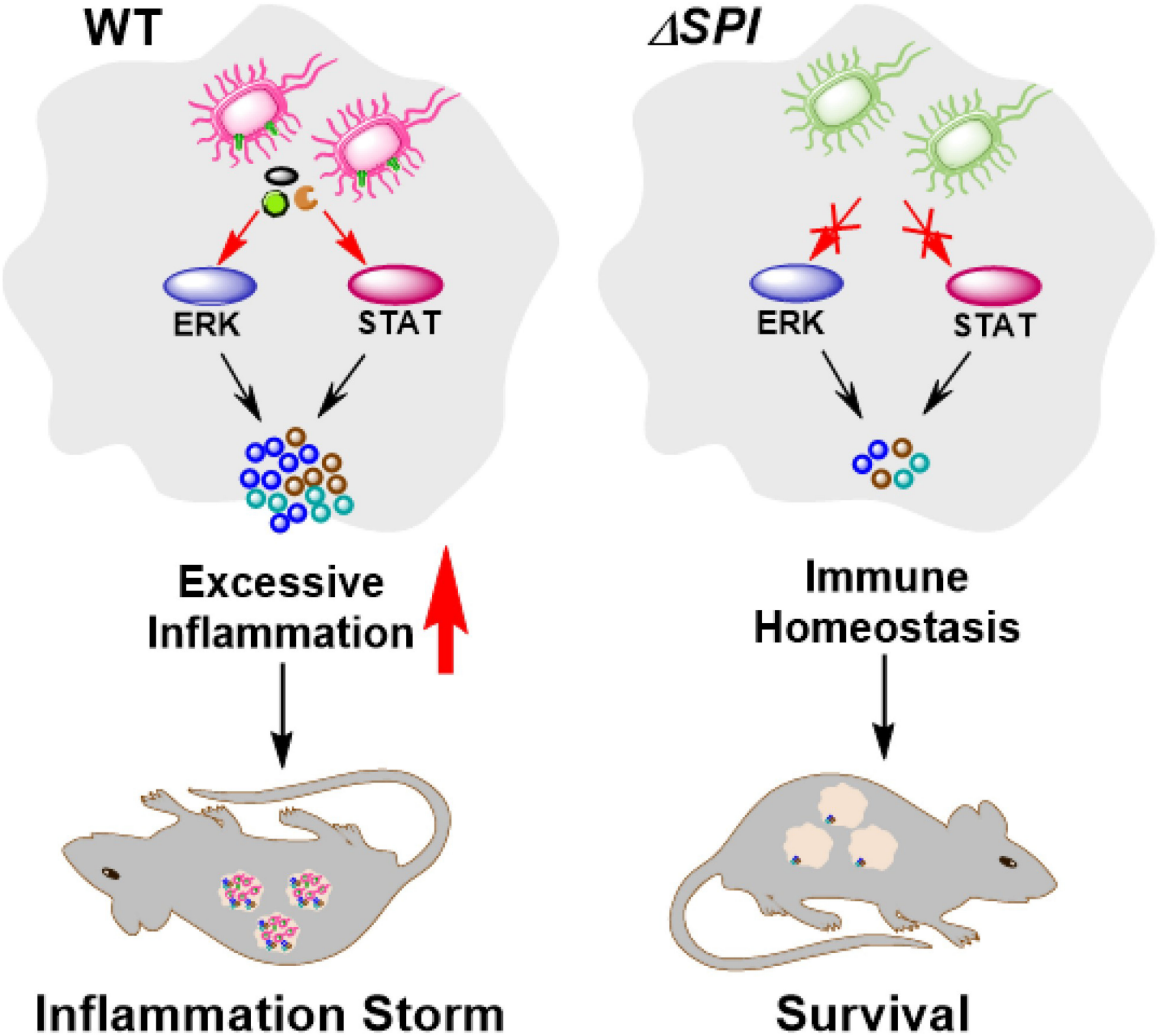
Schematic of *S.* Enteritidis pathopoiesis by SPI-1 and SPI-2 through inducing cytokine storm *via* multi-inflammatory signaling pathways. SPI-1 and SPI-2 activate the ERK- and STAT-mediated inflammatory responses and contribute to severe tissue damages, thus promoting the transmission, intracellular localization and bacterial virulence of *S.* Enteritidis.

To determine the effects of SPI-1 and SPI-2 on the virulence of *S.* Enteritidis, bacterial localization and mouse survival were monitored following *Salmonella* infection. The results show that numbers of bacteria were significantly decreased in liver, spleen and cecum tissues 4 days after challenge with the SPI-1-deficient strain, suggesting that the SPI-1 mutant maintained a moderate level of virulence. However, bacteria could not be detected in *ΔSPI-2*- or *ΔSPI-1/SPI-2*-infected mice, suggesting that strains lacking SPI-2 or SPI-1/SPI-2 were avirulent (**Figures 7A–C**). Furthermore, no deaths were observed when mice were challenged with *ΔSPI-2* or *ΔSPI-1/SPI-2*-deficient strains. In contrast, 20% of animals infected with the same dose of *S.* Enteritidis *ΔSPI-1* died on the sixth day post‐infection and 60% of mice succumbed to infection by the ninth day (**Figure 7D**). These results showed that SPI-1 and SPI-2 enhanced *S.* Enteritidis virulence by causing cytokine storm and promoting bacterial colonization (**Figure 8**).

## 4 Discussion

*S.* Enteritidis causes both gastrointestinal and systemic diseases in a wide range of mammalian hosts, including humans (Jennings et al., 2017). It is estimated that more than 1 billion cases of *Salmonella* gastroenteritis occur every year, resulting in about 3.5 million deaths worldwide (De Masi et al., 2017). *Salmonella* uses two kinds of specialized T3SSs, which facilitate the bacterial colonization and survival within the host. The two T3SSs, encoded by SPI-1 and SPI-2, secrete effectors into the host cell to cause numerous events that result in symptoms of human enteritis (Brown et al., 2005; Chakravortty et al., 2005). The SPI-1 effector SipA activated caspase-3-mediated inflammation in macrophages and epithelial cells (Srikanth et al., 2010; McIntosh et al., 2017). However, *Salmonella* SPI-1 effectors, such as SipB and SipD, reduced the level of nuclear factor κB nuclear translocation to reduce inflammation (Lin et al., 2021). Thus, different effectors in SPIs exhibit various functions with respect to inflammation.

*Salmonella* replication inside macrophages is the key step to induce systemic infection (Gutiérrez et al., 2021). *Salmonella* can induce apoptosis of many types of cells, such as macrophages and dendritic cells (Kiama et al., 2006). Our results show that both SPI-1 and SPI-2 were required for *S.* Enteritidis-induced apoptosis of murine macrophages. Previous studies demonstrated that *Salmonella*-induced apoptosis is SPI-1-dependent (Hueffer and Galán, 2004). However, other studies claimed that apoptosis induced by *S.* Typhimurium could be SPI-1-independent (van der Velden et al., 2000), suggesting various roles of SPI-1 in different serovars. The present study revealed that *S.* Enteritidis SPI-1 and SPI-2 caused elevated LDH release, suggesting that both SPIs enhanced bacterial cytotoxicity, and induced apoptosis following infection of macrophages. These features may help *Salmonella* colonize and spread rapidly in the host.

*Salmonella* infection induces strong inflammatory responses and subsequently makes use of the inflammatory environment generated during the infection process to overcome the resident microbiota and proliferate in the host (Winter et al., 2010; Faber et al., 2017). Inflammation caused by *Salmonella* infection can also disturb the host immune system to enhance bacterial replication in infected cells. Here, we found that inflammatory cytokines and various ISGs mediated by MAPK and STAT signaling pathways were associated with *S.* Enteritidis SPI-1 and SPI-2, suggesting a critical role for *Salmonella* SPI-1 and SPI-2 in cytokine storm activation. Moreover, both SPI-1 and SPI-2 are required for complete induction of inflammation *in vitro*.

We investigated the contribution of SPI-1 and SPI-2 to induction of mouse tissue inflammation and bacterial burden following *S.* Enteritidis infection. Histopathological results revealed focal neutrophil infiltration and necrosis in tissues of mice infected with the *ΔSPI-1* mutant at 4 days post infection. In *ΔSPI-2*-infected mice, however, only steatosis and a few neutrophils were observed. These results indicate that *S.* Enteritidis SPI-2 had a higher capacity to induce inflammatory responses in mice than SPI-1. Moreover, efficient dissemination and colonization of the cecum, liver and spleen requires SPI-2 but not SPI-1 functions. A previous study reported that SPI-1 alone is not enough to cause cecal inflammation in the presence of a sufficient number of intestinal microbiota (Sekirov et al., 2010). We detected the bacterial colonization in the tissues of mice at 4 days following infection, which was the late stage of infection as mice died at 5 days. At this time, mice underwent a systematic infection, and thus high bacterial loads were detected in the livers and spleens. As the *Salmonella* counts in the whole cecum contents could be much higher than that of the cecum itself (Elder et al., 2016), the bacterial load in the washed cecum was relatively lower. In this study, the bacterial load was determined in the washed cecum tissues, not the whole cecum contents. Thus, the bacterial counts from the liver and spleen reached numbers higher than those obtained from the cecum. Similar results have also been reported in other studies. The bacterial loads of livers and spleens were higher than that of colons following challenge with *S.* Enteritidis (van Ampting et al., 2012), ileums following challenge with *S.* Typhimurium (Xu et al., 2018), and cecums following challenge with *S.* Enteritidis (Wang et al., 2022). Collectively, these results indicate that *S.* Enteritidis SPIs, especially SPI-2, could induce inflammatory damage of murine liver and spleen by activating multiple inflammatory signaling pathways.

A previous study showed that a *S.* Typhimurium SPI‐1 mutant strain was unable to damage eukaryotic cells (Riquelme et al., 2016). Another study reported that a *S.* Typhimurium strain with disrupted SPI-1 was not completely attenuated, causing delayed colitis that became obvious at days 3 and 4 following infection (Hapfelmeier et al., 2005). In this study, we found that *S.* Enteritidis SPI-1 deficiency could not induce inflammatory responses or apoptosis *in vitro*. However, the SPI-1-deficient strain contributed to moderate inflammation, tissue damage, and virulence in a mouse model. These results indicate different roles for SPI-1 during *Salmonella* infection *in vitro* and *in vivo*.

Inflammation might be primarily induced by the translocation of various *Salmonella* effector proteins, which trigger the activation of pro-inflammatory signaling. Although both SPI-1 and SPI-2 contain pro-inflammatory and anti-inflammatory effectors, we found that the entire SPI-1 or SPI-2 exhibited a pro-inflammatory phenotype during infection of macrophages and mice. WT *S.* Enteritidis caused significant inflammation of mouse liver and spleen on day 4 following infection, which was attributed to excessive production of inflammatory cytokines. Other studies reported that SPI-1 was insufficient to induce the mouse inflammation according to assessments of intestinal pathology (Sekirov et al., 2010). However, the presence of SPI-2 alone was shown to be sufficient to induce tissue inflammation in a mouse infection model, similar to that induced by *ΔSPI-1/SPI-2*. These results suggest that the virulence of *S.* Enteritidis requires functional SPIs, especially SPI-2, *in vivo*. Notably, *ΔSPI-1* and *ΔSPI-2* mutants were highly attenuated in the macrophage infection assay (by 80% and 70%, respectively). However, SPI-2 played a more important role in colonization and virulence of *S.* Enteritidis in the mouse model. Overall, these results indicate that the complex immune environment *in vivo* may impact the function of different SPIs. Taken together, *S.* Enteritidis promoted its intracellular colonization by activating multiple inflammatory pathways *via* SPI-1 and SPI-2 to contribute to cytokine storm, tissue damage and bacterial virulence.

In conclusion, our findings reveal that SPI-1 and SPI-2 are important virulence factors during *S.* Enteritidis infection *in vitro* and *in vivo*. SPI-1 plays an important role in the invasion of the host cellular cytosol, intracellular survival, and induction of cellular apoptosis *in vitro*, but it does not seem to be required for cytokine storm induction in an *in vivo* infection model. SPI-2 has a more important role than SPI-1 in systemic infection by activating MAPK- and STAT2-mediated inflammation. *S.* Enteritidis contributed to SPI-2-induced cytokine storm and tissue damage to enhance intracellular bacterial replication in the mouse liver and spleen. We identified that both SPIs, especially SPI-2, were required for *S.* Enteritidis colonization and virulence in a mouse model. Our findings increase understanding of the relative roles of SPI-1 and SPI-2 in generation of systemic inflammation and bacterial virulence during *S.* Enteritidis infection.

## Data availability statement

The original contributions presented in the study are included in the article/**Supplementary Material**. Further inquiries can be directed to the corresponding authors.

## Ethics statement

The animal study was reviewed and approved by the Committee on the Ethics of Animal Experiments of Yangzhou University.

## Author contributions

All authors have contributed to the manuscript. DX, XJ and ZP conceived the study. DX designed the experiments, performed the assays and analysed the results. LS and YC performed the experiments and analysed the results. DX, XJ and ZP wrote the paper. All authors read and approved the final manuscript. All authors contributed to the article and approved the submitted version.
