# Supplementary material for: Salmonella Enteritidis activates inflammatory storm via SPI-1 and SPI-2 to promote intracellular proliferation and bacterial virulence
Source: Front Cell Infect Microbiol. 2023 May 30;13:1158888. doi: 10.3389/fcimb.2023.1158888 (PMC10266283; doi:10.3389/fcimb.2023.1158888)
Supplement: Supplementary file 1 [file DataSheet_1.docx]

Supplementary Material

*Salmonella* Enteritidis activates inflammatory storm via SPI-1 and SPI-2 to promote intracellular proliferation and bacterial virulence

Dan Xiong ^1, 2, 3^, Li Song ^1, 2, 3^, Yushan Chen ^1, 2, 3^, Xinan Jiao ^1, 2, 3 *^, Zhiming Pan ^1, 2, 3 *^

*** Correspondence:** Xinan Jiao, [jiao@yzu.edu.cn](mailto:jiao@yzu.edu.cn); Zhiming Pan, zmpan@yzu.edu.cn

**Supplementary Figure**


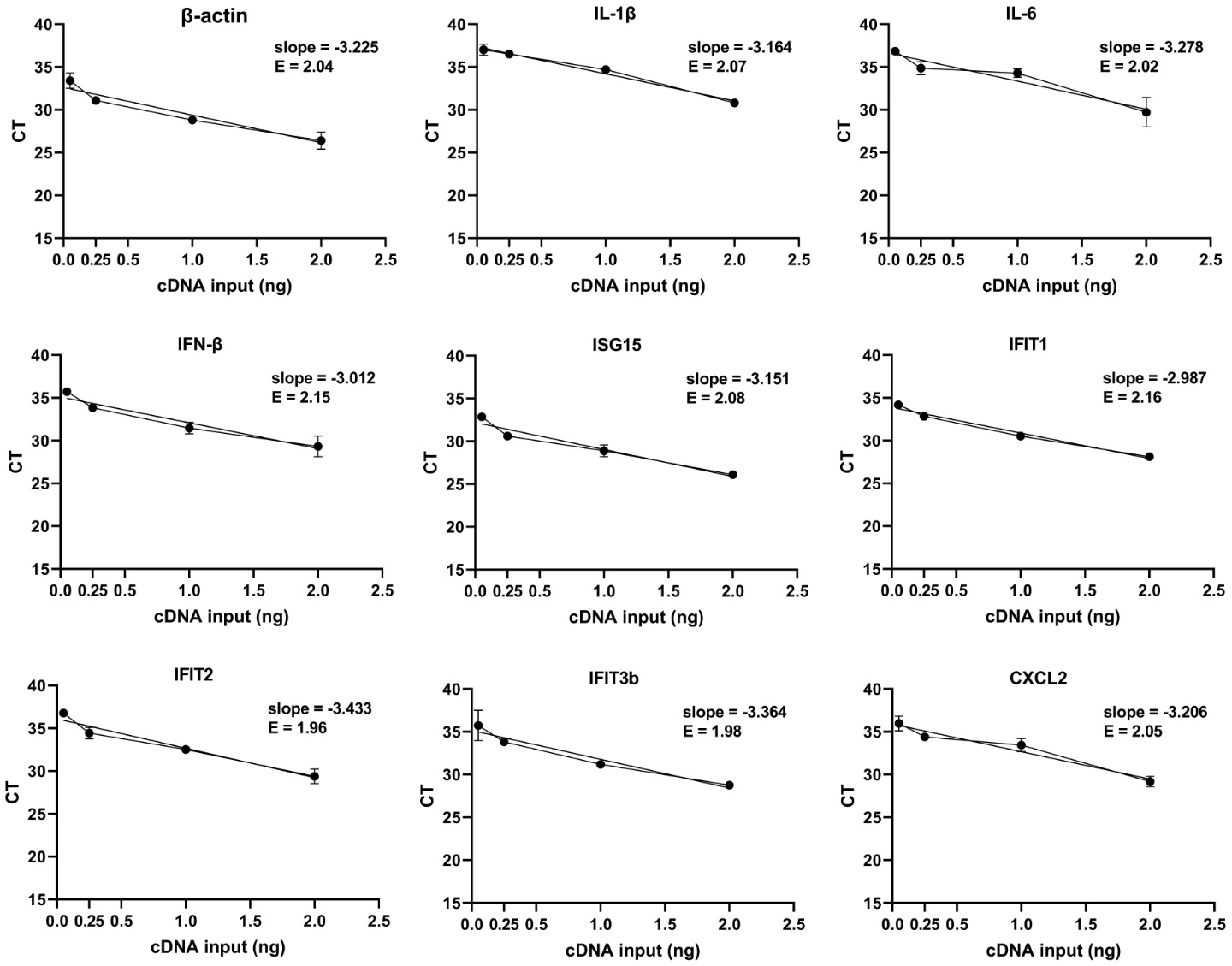


**Supplementary Figure 1.** The real-time PCR efficiencies (*E*) for the reference gene of β-actin and target genes of IL-1β, IL-6, IFN-β, ISG15, IFIT1, IFIT2, IFIT3b and CXCL2 were determined using the diluted cDNA templates, and calculated based on the equation: *E* = 10^[–1/slope]^ (Rasmussen, 2001).

**Supplementary Table 1 Bacterial strains and plasmids used in this study.**

| **Strain or plasmid** | **Description** | **References** |
| --- | --- | --- |
| C50041 WT | *Salmonella* *enterica* serovar  Enteritidis CMCC50041 | (Xiong et al., 2019) |
| *ΔSPI-1* | C50041 *ΔSPI-1* (deficient strain) | (Xiong et al., 2019) |
| *ΔSPI-2* | C50041 *ΔSPI-2* (deficient strain) | (Xiong et al., 2019) |
| *ΔSPI-1/SPI-2* | C50041 *ΔSPI-1/SPI-2* (deficient strain) | this study |
| pKD3 | Template plasmid; FRT-*aphT*-FRT  (containing chloramphenicol resistance gene) | (Datsenko and Wanner, 2000) |
| pKD46 | Red recombinase expression plasmid  *bla*p*BAD* *gam* *bet* *exo*pSC101 *ori*TS  (containing ampicillin resistance gene) | (Datsenko and Wanner, 2000) |
| pCP20 | FLP recombinase expression plasmid | (Datsenko and Wanner, 2000) |

**Supplementary Table 2 Primer sequences used for the construction of *S.* Enteritidis *ΔSPI-1*, *ΔSPI-2*, and the *ΔSPI-1/SPI-2* deficient strains in this study.**

| **Primer name** | **Primer sequence (5′→3′)** | **Application** |
| --- | --- | --- |
| *ΔSPI-1* F | GCTGTCGCGTATGAAGCGATTGGGTATTGATAAAGACGCGTTAGCGTAAGTGTAGGCTGGAGCTGCTTC | Construction of C50041 *ΔSPI-1* deficient strain |
| *ΔSPI-1* R | ATATGGTCTTAATTATATCATGATGAGTTCAGCCAACGGTGATATGGCCCATATGAATATCCTCCTTAG |  |
| *ΔSPI-2* F | TCCAGGACGCGTGGTATTGGCATATCGGTGGGATGATAGCCAAGACAAACGTGTAGGCTGGAGCTGCTTC | Construction of C50041 *ΔSPI-2* deficient strain |
| *ΔSPI-2* R | TGCCTCGCTCTAAGGATAGGTGACATCGAAAGAGCGTGCAGAGGAATGTGCATATGAATATCCTCCTTAG |  |
| DetSPI-1 F | CCAACGCATAATCTGCTTCA | Identification of C50041 *ΔSPI-1* deficient strain |
| DetSPI-1 R | CCGGAAAGGTGCTGATAAAA |  |
| DetSPI-2 F | GATTACGCACTACCGCAACA | Identification of C50041 *ΔSPI-2* deficient strain |
| DetSPI-2 R | ATTTCAGCTGATTGCGCTTT |  |
| *stn* F | TATTTTGCACCACAGCCAGC | Identification of *Salmonella* genus |
| *stn* R | CGACCGCGTTATCATCACTG |  |

**Supplementary Table 3 Primer sequences used for the detection of inflammatory cytokines and various ISGs by the quantitative real-time PCR in this study.**

| **Gene** | **Forward primer**  **(5'-3')** | **Reverse primer**  **(5'-3')** | **Product size (bp)** | **Accession no.** |
| --- | --- | --- | --- | --- |
| IL-1β | gcccatcctctgtgactcat | aggccacaggtattttgtcg | 230 | NM_008361.4 |
| IL-6 | agttgccttcttgggactga | tccacgatttcccagagaac | 159 | NM_031168.2 |
| CXCL2 | agtgaactgcgctgtcaatg | ttcagggtcaaggcaaactt | 153 | NM_009140.2 |
| IFN-β | ccctatggagatgacggaga | ctgtctgctggtggagttca | 161 | NM_010510.1 |
| ISG15 | aagaagcagattgcccagaa | tctgcgtcagaaagacctca | 217 | NM_015783.3 |
| IFIT1 | aggctggagtgtgctgagat | tctggatttaaccggacagc | 224 | NM_008331.3 |
| IFIT2 | caccttcggtatggcaactt | gcaaggcctcagaatcagac | 181 | NM_008332.3 |
| IFIT3b | cgagcaaaaatgtgctttga | gctccccttcagcttcttct | 190 | NM_001005858.3 |
| β-actin | agccatgtacgtagccatcc | ctctcagctgtggtggtgaa | 228 | NM_007393.5 |

**Supplementary Table 4 Histopathology score used in this study.**

| **Score** | **Neutrophils** | **Necrosis** |
| --- | --- | --- |
| 0 | no lesion | no lesion |
| 1 | mild focal to multifocal neutrophilic infiltration | mild focal necrosis/mild diffuse/cell swelling |
| 2 | mild to moderate muiltifocal neutrophilic infiltration | mild to moderate multifocal necrosis/ moderate diffuse/cell swelling |
| 3 | moderate multifocal neutrophilic infiltration | moderate multifocal necrosis/severe diffuse/cell swelling |
| 4 | severe multifocal to coalescent neutrophilic infiltration | severe multifocal to coalescent necrosis |

The scores for neutrophils were defined as follows: 0, < 5 neutrophils/high power field; 1, 5–20 neutrophils/high power field; 2, 21–60 neutrophils/high power field; 3, 61–100 neutrophils/high power field; and 4, > 100 neutrophils/high power field.

The scores for necrosis were defined as follows: 0, no necrosis/high power field; 1, < 10% necrosis/high power field; 2, 11%–40% necrosis/high power field; 3, 41%–70% necrosis/high power field; and 4, > 71% necrosis/high power field.

The scores for cell swelling were defined as follows: 0, < 105% diameter/high power field; 1, 106%–125% diameter/high power field; 2, 126%–150% diameter/high power field; and 3, > 151% diameter/high power field. Shown is the diameter ratio of swelling cells compared to normal cells. Cell diameter was determined by quantitative microscopy and represents the average of 20 cells per field.
